# Understanding the molecular basis of anorexia and tissue wasting in cancer cachexia

**DOI:** 10.1038/s12276-022-00752-w

**Published:** 2022-04-06

**Authors:** Eunbyul Yeom, Kweon Yu

**Affiliations:** 1 Metabolism and Neurophysiology Research Group, Disease Target Structure Research Center, KRIBB, Daejeon, 34141 Korea; 2 School of Life Sciences, College of Natural Sciences, Kyungpook National University, Daegu, 41566 Korea; 3 School of Life Sciences, BK21 FOUR KNU Creative BioResearch Group, Kyungpook National University, Daegu, 41566 Korea; 4 Department of Functional Genomics, UST, Daejeon, Korea

**Keywords:** Cancer metabolism, Cancer metabolism

## Abstract

Cancer cachexia syndrome is a major cause of morbidity and mortality in cancer patients in the advanced stage. It is a devastating disorder characterized by nutritional impairment, weakness, and wasting, and it affects treatment success and quality of life. Two major symptoms of cancer cachexia are anorexia and weight loss. Weight loss in cachexia is not reversed through increased food intake, suggesting that anorexia and weight loss in cancer patients are regulated by independent molecular mechanisms. Although the wasting phenotype mostly occurs in skeletal muscle and adipose tissue, other organs, such as the brain, liver, pancreas, heart, and gut, are also involved in cachexia. Thus, cachexia is a multiorgan syndrome. Although the molecular basis of cancer cachexia-induced weight loss is known, the mechanism underlying anorexia is poorly understood. Here, we highlight our recent discovery of a new anorexia mechanism by which a tumor-derived humoral factor induces cancer anorexia by regulating feeding-related neuropeptide hormones in the brain. Furthermore, we elucidated the process through which anorexia precedes tissue wasting in cachexia. This review article aims to provide an overview of the key molecular mechanisms of anorexia and tissue wasting caused by cancer cachexia.

## Cancer: When tumors cause weight loss

Tumors can release factors that cause anorexia and weight loss in cancer patients, negatively impacting quality of life and treatment success. Patients with this condition, known as cachexia, can lose their appetite and be unable to gain weight even if they eat more. Although cancer cachexia directly causes the death of up to 20% of cancer patients, the mechanisms are poorly understood. Eunbyul Yeom and Kweon Yu at The Korea Research Institute of Bioscience and Biotechnology, Daejon, South Korea have reviewed the causes of cancer cachexia, highlighting their recent discovery that tumors produce a signaling molecule that induces anorexia by disrupting hunger signaling in the brain. Improving our understanding of the mechanisms underlying cancer cachexia may help in development of treatments.

## Introduction

Cancer cachexia is defined as a multifactorial metabolic syndrome occurring in advanced cancer patients that negatively affects quality of life and leads to early death^1–5^. Anorexia and weight loss are two major symptoms of cancer cachexia^6^. Weight loss occurs because of the loss of skeletal muscle and fat tissue, and cancer anorexia is caused by a failure of appetite signals^2,7^. Cancer cachexia not only decreases the quality of life of cancer patients but also has negative effects during the chemotherapy process^8^. Therefore, understanding the underlying molecular mechanisms is critical to ensure an accurate diagnosis of cancer cachexia and to design treatments for cancer patients. Cancer cachexia is detected in more than 80% of gastrointestinal or pancreatic cancer patients and is less common in lymphoma or breast cancer patients^9,10^. Anorexia and weight loss induced by cancer cachexia are the direct causes of death in up to 20% of all cancer patients^11,12^. The failure of appetite signaling caused by cancer anorexia differs from malnutrition or hunger; it is caused by metabolic abnormalities such as increased basal metabolism, decreased fat production, increased fat breakdown, and increased muscle protein breakdown^13^. Anorexia can negatively impact cancer patients through weight loss and adipose tissue wasting, but weight loss does not always occur with cancer anorexia^14^. In addition, the weight loss is not recovered through increased food intake, suggesting that anorexia and weight loss in cancer cachexia patients are controlled by independent molecular mechanisms^1,2,15^.

The major cause of cancer cachexia is the deterioration of normal tissue function caused by inflammatory cytokines, which are released in excessive amounts by tumors^16^. Tumor growth triggers an inflammatory response around the tumor, which induces the secretion of inflammatory cytokines, such as tumor necrosis factor-α (TNF-α), interleukin (IL)-6, and IL-1β^17,18^. These secreted tumor-derived humoral cytokines play an essential role in interorgan/tissue communication. The interorgan communication that underlies the systemic coordination and integration between organs and tissues is important under normal and pathological conditions, such as cancer and metabolic syndrome^19^. Tumors elicit an inflammatory response that leads to metabolic dysfunction in muscle and fat, as well as in the brain, by causing an inflammatory cytokine burst^20,21^. Recent data indicate that various factors other than inflammatory cytokines are produced and secreted in a cancer cachexia model. In a cancer model, the secretion of transforming growth factor-beta (TGF-β) and parathyroid-hormone related protein (PTHrP) causes the cachexia phenotype by inducing an imbalance of muscle tissue and fat metabolism^22–24^. Exposure to a neutralizing antibody against PTHrP alleviates cancer cachexia^25^.

In a statistical analysis of 1307 cancer patients, cachexia was divided according to severity into the pre-cachexia stage, with a weight loss of <10%, and the cachexia stage, with a weight loss of >10%. In addition to weight loss, loss of appetite, fatigue, and early satiety are important factors involved in the pre-cachexia stage and the cachexia stage^26^. However, the relationship between cancer anorexia and weight loss during cancer cachexia remains unclear. Furthermore, the pathological mechanisms, criteria for diagnosis, and applicable blood biomarkers for cancer cachexic patients are not yet available. This review examines the molecular mechanisms underlying cancer cachexia by analyzing the two major processes, namely, cancer anorexia and tissue wasting. In addition, we examined the relationship between anorexia and wasting in cancer cachexia.

## Cancer anorexia

Anorexia (loss of appetite) is a major symptom of cachexia that develops in most cancer patients^27^. Among the multiple causes of cancer anorexia^28^, the primary cause is the increase in substances released by the tumor, such as pro-inflammatory cytokines or lactate^21^. Alternatively, there are many peripheral causes of anorexia, including dysphagia, alterations in gastrointestinal functions, hypoxia, alterations in nutrients by the tumor, and alterations in food intake due to the release of peripheral hormones^21^. Chemotherapy also causes anorexia. Chemotherapy peripherally affects taste perception and may cause nausea, vomiting, and dysgeusia. However, the underlying mechanism remains unclear^29^. In addition to these peripheral causes, the central cause of anorexia may be related to multiple alterations in various neurotransmitters or neuropeptides, which can lead to depression, pain, and decreased appetite^21^.

Cytokines are peptide hormones released from tumor tissues or the immune system. Pro-inflammatory cytokines, such as TNFα, IL-1, and IL-6, are increased in tumors^30,31^. Studies show that these cytokines are the direct cause of reduced food intake in cancer patients^32–34^. IL-1 is secreted by T cells and macrophages, and it is the most potent anorectic factor. IL-1 can decrease the size, duration, and frequency of meal intake^35^, and antibodies against IL-1 restore food intake in tumor-bearing mice^36^. TNFα produced by macrophages and monocytes is increased in tumors in mice and induces anorexia via central and peripheral effects^37–39^.

We recently reported that a new type of cytokine, Dilp8/INSL3, induces anorexia in cancer patients^40^. *Drosophila* Dilp8, the mammalian homolog of INSL3, is released from tumor tissues and systemically controls the expression of feeding-associated neuropeptides through the Lgr3/Lgr8 receptor in the brain. We demonstrated that the regulation of INSL3 in cancer anorexia is conserved in mice. The study showed that the secretion of INSL3 into cell culture medium and serum INSL3 levels are increased in a mouse cancer model and human cancer patients, respectively. Direct injection of INSL3 into the brain reduces food intake in wild-type mice, whereas no changes are observed in response to a peripheral injection of INSL3^40^. The peripheral injection of INSL3 in LLC tumor-bearing mice initially showed no changes in food intake, but these mice ultimately developed anorexia. These findings suggest that various systemic changes are necessary for INSL3 delivery to the hypothalamus. Recent studies of cancer cachexia using the fly cancer model reported a dramatic increase in the expression of three tumor-derived cytokines, Dilp8, ImpL2, and Upd2^40–42^. ImpL2 is a *Drosophila* homolog of mammalian insulin-growth factor binding protein (IGFBP), and it causes organ wasting independent of food consumption. Another factor, Upd2, does not cause organ wasting or anorexia in flies, whereas mouse IL-6 (Upd2 ortholog) induces white adipose tissue (WAT) browning under cachexia conditions^43^. Tumor-derived IL-6/Upd2 induces noncell-autonomous autophagy around tumor tissues^44^.

In the central nervous system, tumors cause changes in neurotransmitters and neuropeptides that alter feeding. The hypothalamus, which is a critical region for cachexia/anorexia development, regulates both food intake and body energy expenditure^45^. At the central level of the hypothalamus, peripheral signals regulate orexigenic factors, such as neuropeptide Y (NPY) and agouti-related protein (AgRP), through a food intake-promoting axis or anorexigenic factors, such as pro-opiomelanocortin (POMC) precursor. POMC is involved in the production of the melanocyte-stimulating hormone α-MSH^46^. The main appetite-stimulating neuropeptides NPY and AgRP originate from the same arcuate nucleus neurons near the hypothalamic median eminence^46,47^. NPY binds to postsynaptic Y1 and Y5 receptors, which release AgRP^46^. In a parallel system, POMC neurons from the arcuate nucleus innervate both the paraventricular and lateral hypothalamus. POMC reduces food intake by binding to postsynaptic melanocortin receptor 4, which releases secondary effectors such as thyrotropin-releasing hormone, corticotropin-releasing hormone (CRH), and oxytocin^48,49^. In addition, leptin is the major appetite-suppressing hormone that activates POMC gene expression and inhibits NPY expression. Leptin is produced by WAT and exerts anorexigenic effects through CRH^50,51^. However, there is currently no evidence supporting a role for leptin in cancer anorexia. Although the circulating leptin level in cancer cachexia patients is decreased, it is not associated with a compensatory increase in food intake^52^.

In recent work from our group, we showed that the anorexigenic factor nesfatin-1 is involved in inducing cancer anorexia^40^. The Nesfatin-1 peptide consists of 82 amino acids produced from the nucleobindin 2 (NUCB2) precursor. It is expressed in the hypothalamic region and reduces food intake in mammals^53^. The NUCB2 precursor protein is expressed in the hypothalamic region, including the supraoptic nucleus, lateral hypothalamic area, arcuate nucleus, paraventricular nucleus^53^, and parabranchial nucleus (PBN)^54^. Recent findings indicate that calcitonin gene-related peptide-positive neurons in the PBN region contribute to cancer-induced appetite suppression in LLC tumor-implanted mice^55^. We validated the anorectic effect of NUCB1 (NUCB2 homolog in *Drosophila*) in the *Drosophila* system and proposed that NUCB1-expressing neurons in the brain are important for anorexigenic regulation under normal and malignant conditions. However, the detailed function of the Nesfatin-1 receptor remains to be discovered. Thus, further studies are needed to examine the receptor for Nesfatin-1 and to elucidate the precise mechanism by which Nesfatin-1 induces anorexia.

## Tissue wasting in skeletal muscle and adipose tissue

Cancer cachexia is considered a disorder of energy balance that occurs when energy intake decreases and/or when energy expenditure increases^56–59^. The balance of energy expenditure can depend on the type and stage of tumors. Increased energy expenditure is a possible cause of wasting syndrome that leads to involuntary weight loss. One example of a major molecular mechanism that contributes to energy expenditure is the “Cori cycle”. The Warburg effect results in the production of an excess amount of lactate by tumors due to anaerobic glycolysis. The Cori cycle mediates the hepatic uptake of excess lactate produced by tumors and its conversion to glucose. It then returns to the circulation and is reused for glycolysis. This inefficient recycling of lactate requires high energy because the conversion of glucose into lactate generates less ATP than the amount needed to produce glucose from lactate^60^. Another wasting mechanism identified in a mouse cancer model is decreased mitochondrial ATP synthesis in skeletal muscles^61^. In the skeletal muscle mitochondria of cancer patients, decreased oxidative capacity and membrane fluidity impair mitochondrial function^62^. However, the biochemical mechanisms of wasting remain elusive.

Wasting during cancer cachexia occurs mostly in skeletal muscle and adipose tissue. Skeletal muscle is an essential organ for various biological processes, including body movement and respiration. The maintenance of muscle homeostasis requires a balance between protein synthesis and degradation, and a decrease in protein synthesis or excessive degradation results in muscle wasting^63^. During tumor progression, cytokines or other factors that regulate the anabolic and catabolic system network are disrupted^64^. Studies suggest that the circulating level of the anabolic factor insulin-like growth factor-1 (IGF-1) is decreased, and insulin resistance occurs in the cancer cachexia model^65–70^. In contrast, the production of pro-cachexic or pro-inflammatory factors, such as TNF-α^71,72^ and IL-1^73–76^, is increased, thereby promoting catabolism. These cytokines are involved in two well-known signaling pathways: the nuclear factor-κB (NF-κB) and p38 MAPK pathways^77^. In the NF-κB pathway, inflammatory mediators, such as myostatin or proteolysis-inducing factors, cause the transcriptional upregulation of genes encoding ubiquitin ligases (muscle RING finger-containing protein 1 (MURF-1) and muscle atrophy F-box protein). These ligases mediate proteolysis of myofibrillar proteins and inhibit protein synthesis^77^. Accordingly, inhibition of NF-κB by blocking the upregulation of MURF-1 significantly decreases muscle weight loss in a cancer cachexia model^78,79^. In the p38 MAPK pathway, mediators activate the p38 and MAPK cascades, which increase the activity of caspases, leading to apoptosis^77^.

Recent studies have examined the mechanism of insulin resistance using *Drosophila* cancer models^41,42^. In these cachexia models, the expression of ImpL2, the *Drosophila* homolog of mammalian IGFBP, is increased in tumor tissues, which disrupts both insulin and IGF-1 signaling. Secretion of ImpL2 by tumor tissues directly promotes insulin resistance in peripheral organs, such as muscle, fat, and ovaries, and ultimately causes systemic wasting^41,42^. In addition, direct exposure of C2C12 muscle cells to IGFBP3 induces muscle cell wasting, and the wasting effect is improved by IGFBP3 knockdown or IGFBP3 antibody neutralization^80^.

Growth differentiation factor-15 (GDF15), a TGF-β superfamily member, was recently highlighted as a biomarker for the early diagnosis of cachexia^81–84^. Antibody-mediated inhibition of the GDF15 receptor reverses cancer cachexia in mice, suggesting a novel function of the inflammatory growth factor GDF15^85^. This study identified a correlation between the circulating level of GDF15 and cachexia and showed that GDF15-induced body weight loss is mediated by the GDNF family receptor-A-like (GFRAL)-Ret proto-oncogene (RET) signaling complex in brainstem neurons^85^. In addition, a study showed that activation of the GFRAL-RET pathway induces the expression of genes involved in lipid metabolism and adipose tissues, and GDF15-mediated weight loss is prevented in adipose triglyceride lipase knockout mice^85^. This breakthrough study showed that inhibition of the GDF15-GFRAL pathway is a novel strategy for the treatment of cachexia in a mouse model, and this approach is currently being evaluated in phase I clinical trials.

In parallel with skeletal muscle loss, adipose tissue wasting commonly accompanies cancer cachexia. WAT wasting occurs through three different processes^86^. First, lipolytic activity is increased, leading to the release of high levels of free fatty acids and glycerol^86^. This event is involved in the activation of hormone-sensitive lipase. This lipase stimulates lipolysis and is related to the increased circulation of the lipid mobilization-promoting adipokine ZAG in adipose tissue^71,87^. Second, decreased activity of lipoprotein lipase, which cleaves triacylglycerol into glycerol and fatty acids, disrupts lipid uptake^86^. Last, de novo lipogenesis in WAT is reduced in cancer cachectic patients or mice; thus, triacylglycerol synthesis and lipid deposition are decreased^86^. WAT changes to brown adipose tissue (also called “browning”) during cancer cachexia^24,43^. The browning process is caused by high expression of uncoupling protein 1 (UCP1) in mitochondria, which switches the electrochemical gradient from ATP synthesis to thermogenesis^88^. This results in increased lipid mobilization and energy expenditure, which are common in cancer cachectic patients^24^. Several factors, such as IL-6 and PTHrP, which are derived from tumor or immune cells, are involved in the regulation of UCP1^24,25^.

The wasting of skeletal muscle and adipose tissue is a critical factor that contributes to cancer cachexia by stimulating lipolysis and increasing energy expenditure. Additional features that are characteristic of cancer cachexia and occur in other organ systems will be discussed below.

## Cancer anorexia precedes wasting

**Fig. 1:**
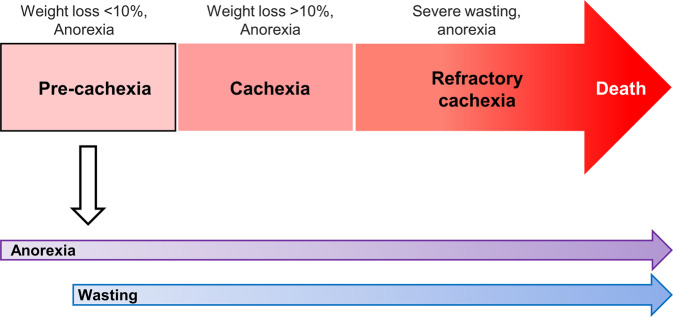
Stages of cancer cachexia, anorexia, and wasting in the pre-cachexia stage. Cancer cachexia can be divided into three clinical stages: pre-cachexia, cachexia, and refractory cachexia. During the pre-cachexia stage, both anorexia and weight loss occur independently through separate mechanisms. More specifically, anorexia precedes the onset of wasting.

Cancer cachexia can be divided into three clinical stages according to weight loss: pre-cachexia (<10% weight loss), cachexia (≥10% weight loss), and refractory cachexia^2^ (Fig. 1). The frequency, penetrance, and severity of cachexia are highly variable and depend on the type and stage of tumors. Specific biomarkers for the diagnosis or treatment of early-stage cachexia have not been identified to date. Anorexia and weight loss are regulated by independent mechanisms, and cancer induces cachexia with body weight loss or the wasting phenotype independent of food intake. Despite several attempts to classify and categorize the stages of cachexia in cancer patients, there is limited information on the relationship between weight loss and anorexia, including the preference of the occurrence of the two symptoms.

In a recent study, in addition to elucidating the mechanism of cancer anorexia in both *Drosophila* and mammalian systems, we discovered that the process of anorexia precedes the onset of wasting^40^ (Fig. 1). Overexpression of the oncogene *yki* in the *Drosophila* cancer model demonstrated that the anorexia phenotype appeared early, as it started at 5 days. This finding is consistent with the fact that most cancer patients show a loss of appetite at the pre-cachexia stage, whereas the organ-wasting phenotype appears at 15–20 days. This finding was demonstrated in the *Drosophila* cancer model as well as in the mouse cancer model. In C26 tumor-bearing mice, anorexia induced by INSL3 secretion started on Day 11, although no body weight changes were observed in this time period. Alteration of body weight and muscle/fat atrophy markers occurs at 2–3 weeks after tumor cell implantation, suggesting that anorexia develops prior to the process of wasting in a mouse cancer model. Although the inhibition of Dilp8 does not restore the cancer-induced organ-wasting phenotype^41^, Dilp8 inhibition rescues the cancer anorexia phenotype^40^. These findings suggest that Dilp8 and ImpL2 play critical roles in regulating cancer anorexia and wasting, respectively.

The significance of identifying INSL3/Dilp8 as a new tumor-derived anorexia factor was described in a recent commentary by Wang et al.^89^. These findings highlight our discovery of a new tumor-derived factor in the brain that induces anorexia. Previous studies have focused on cancer-induced cachexia in advanced or metastatic stages. As noted by Wang et al.^89^ the finding that INSL3 functions at early stages indicates that cancer patients exhibit anorexia ahead of involuntary weight loss. In addition, recent evidence suggests that other systemic effects induced by cancer, such as premetastatic niche formation, occur in the early stages of cancer before progression to metastatic disease^90^. Further studies elucidating the relationship between anorexia and other systemic effects will improve our understanding of the early stages of cancer progression^89^. Therefore, targeting tumor-derived factors related to anorexia or other systemic effects in the early stages could be beneficial for preventing or treating cancer cachexia.

## Cancer cachexia as a multiorgan syndrome

**Fig. 2:**
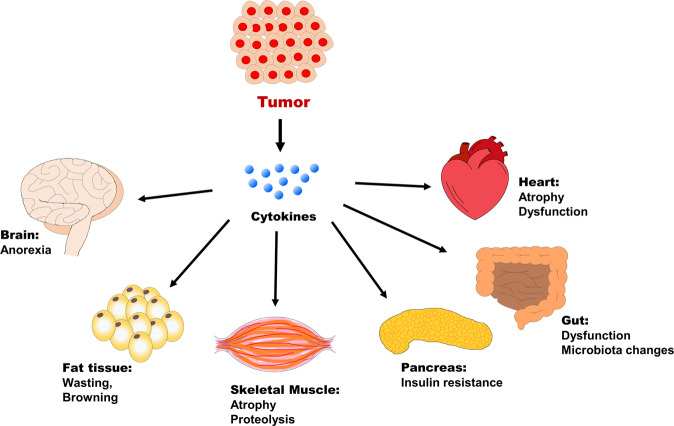
The schematic illustration represents the major organs affected during the progression of cancer cachexia. Tumor cells secrete pro-inflammatory cytokines, which induce systemic changes in multiple tissues. In addition to the major symptoms of anorexia and wasting, other organs are affected by the cachectic process. This involves abnormalities of the heart, gut, and pancreatic functions.

Weight loss that occurs in skeletal muscles accounts for >40% of the cachexia phenotype. However, recent studies suggest that other tissues, such as adipose tissue, brain, liver, pancreas, heart, and gut, are also involved in cachexia development and are related to muscle wasting (Fig. 2). In addition to the involvement of adipose tissue and the brain in the cachectic process, recent evidence suggests that the gut microbiota is involved in cancer cachexia^91,92^. In the proposed gut microbiota and skeletal muscle axis, the gut microbiota generates metabolites that are delivered to skeletal muscle, leading to increased energy expenditure in muscle cells^93,94^.

Another link between the gastrointestinal tract and skeletal muscle wasting is ghrelin. Ghrelin is a 28-amino acid peptide that is mainly secreted by the stomach and increases food intake through a nitric oxide-dependent mechanism^95^. Although circulating ghrelin levels are elevated in cancer patients, “ghrelin resistance” is also observed in cancer patients^69,96^. However, the administration of a long-acting ghrelin analog increases food intake and weight gain in tumor-implanted rats^97^. Ghrelin also directly influences muscle cells by blocking the increased protein degradation that is promoted by catabolic cytokines^98^. Based on its orexigenic properties, ghrelin has recently been investigated in clinical studies as a treatment for patients with anorexia or those undergoing chemotherapy. Studies show that ghrelin increases food intake and meal appreciation in cancer anorexia patients^99^. In patients undergoing chemotherapy, ghrelin improves food intake and appetite, thereby alleviating the effects of anorexia and nausea^100^. Glucagon-like peptide 1 (GLP-1) is another gut hormone that promotes insulin secretion and has been extensively explored for the treatment of metabolic diseases, such as obesity and type 2 diabetes. Despite its well-known role in metabolism, the link between GLP-1 and cachexia has not been studied in detail. Cachexia patients often show changes in glucose homeostasis and insulin sensitivity. The GLP-1 agonist and insulin sensitizer exendin-4 partially rescued cachexia in tumor-bearing rats^101^. Additional recent evidence suggests that brainstem GLP-1 signaling contributes to body weight loss and lean/fat mass in a cachexia rat model^102^. These findings suggest that gut hormones are good candidates for the treatment of cancer cachexia.

Glucose intolerance was first identified in cancer patients in 1919. Higher endogenous glucose production with increased gluconeogenesis is associated with cancer cachexia^103^. Insulin resistance is another common feature of cancer cachexia, as shown in multiple animal models. In pancreatic islets of Langerhans isolated from carcinoma-bearing rats, insulin secretion is decreased in response to glucose stimulation, indicating impaired insulin sensitivity^104^. Despite decreased blood glucose levels in adenocarcinoma-bearing mice, insulin sensitivity is decreased, inducing insulin resistance^66^. Given the role of insulin in the maintenance of skeletal muscle, this evidence supports the involvement of insulin resistance in the development of tissue wasting in cancer cachexia patients.

Cardiac dysfunction, which occurs in human cancer patients and mouse cancer models, is closely associated with muscle wasting. Tumor-bearing rats show decreased heart weight and deterioration of the function of the heart, eventually leading to heart failure^105^. Increasing evidence indicates that cardiac atrophy is associated with growth inhibition by activating ACTRIIB, which is stimulated by TGF-β family ligands such as myostatin, activin, and growth/differentiation factor 11 (GDF11)^106^. Another study of heart alterations in cachexia found that cardiac oxygen consumption is increased in a mouse cancer model^107^. This increased oxygen consumption can be linked to increased energy expenditure by causing inefficient energy generation. Therefore, the elevated heart rate often seen in cancer patients could be a critical parameter of the risk of cancer death.

## Concluding remarks

In this review, we discussed the key mechanisms of anorexia and tissue wasting induced by cancer cachexia. Despite more than 10 years of research into the mechanisms underlying cachexia, specific biomarkers for the diagnosis or treatment of this condition remain unavailable. Early detection of cancer cachexia syndrome increases treatment efficiency. Tumor-implanted rodent models are used for the discovery of the molecular mechanisms of cachexia. However, because the anorexia and wasting phenotypes of cachexia have different penetrance among cancer types, using different strains or different cancer models is critical to examine the variety of cachectic processes observed in cancer patients. In addition, because cachexia is a multiorgan syndrome that affects different tissues simultaneously, therapeutic strategies should be designed to target multiple organs.

Although cancer cachexia syndrome occurs independently from chemotherapy, there is evidence that chemotherapy can promote cachexia development, including anorexia^21^. However, the molecular mechanism underlying the effect of chemotherapy on inducing anorexia remains largely unknown. Therefore, it will be interesting to analyze whether known tumor-derived factors, such as INSL3 and IGFBP, are also involved in chemotherapy-induced cachexia.
